# Case Report: Differences in self-selected pacing in 20, 40, and 60 ironman-distance triathlons: a case study

**DOI:** 10.3389/fspor.2024.1155844

**Published:** 2024-09-16

**Authors:** Beat Knechtle, Ivan Cuk, Marilia Santos Andrade, Pantelis T. Nikolaidis, Katja Weiss, Pedro Forte, Mabliny Thuany

**Affiliations:** ^1^ Medbase St. Gallen Am Vadianplatz, St. Gallen, Switzerland; ^2^ Institute of Primary Care, University Hospital Zurich, Zurich, Switzerland; ^3^ Faculty of Sport and Physical Education, University of Belgrade, Belgrade, Serbia; ^4^ Department of Physiology, Federal University of São Paulo, São Paulo, Brazil; ^5^ School Health and Caring Sciences, University of West Attica, Athens, Greece; ^6^ Research Center for Active Living and Wellbeing, Instituto Politécnico de Bragança, Bragança, Portugal; ^7^ Research Center in Sports, Health and Human Development, Covilhã, Portugal; ^8^ Department of Sports Sciences, Instituto Politécnico de Bragança, Bragança, Portugal; ^9^ Centre of Research, Education, Innovation and Intervention in Sport, Faculty of Sports, University of Porto, Porto, Portugal

**Keywords:** swimming, cycling, running, multi-stage triathlon, pacing

## Abstract

**Background:**

Triathletes are pushing their limits in multi-stage Ironman-distance triathlons. In the present case study, we investigated the pacing during 20, 40, and 60 Ironman-distance triathlons in 20, 40, and 60 days, respectively, of one professional IRONMAN® triathlete.

**Case study:**

Event 1 (20 Ironman-distance triathlons in 20 days), Event 2 (40 Ironman-distance triathlons in 40 days), and Event 3 (60 Ironman-distance triathlons in 60 days) were analyzed by discipline (swimming, cycling, running, and overall event time), by Deca intervals (10 days of consecutive Ironman-distance triathlons) and additional data (sleep duration, body mass, heart rate in cycling and running). To test differences between Events and Deca intervals within the same discipline, *T*-tests (2 groups) or one-way ANOVAs (3 or more groups) were used.

**Results:**

Swimming splits were fastest in Event 1, (*ii*) cycling and running splits were fastest in both Event 2 and 3, (*iii*) overall speed was fastest in Event 3, (*iv*) sleep duration increased during Event 2 but decreased in Event 3, (*v*) body mass decreased in Event 2, but increased in Event 3 and (*vi*) heart rate during cycling was similar in both Event 2 and 3. In contrast, heart rate during running was greater in Event 3.

**Conclusion:**

In a professional IRONMAN® triathlete finishing 20, 40, and 60 Ironman-distance triathlons in 20, 40, and 60 days, respectively, split performances and both anthropometrical and physiological changes such as body mass and heart rate differed depending upon the duration of the events.

## Introduction

In a triathlon race, performance depends upon different factors, including race strategy (1) and pacing (2). The race strategy or race tactics in triathlon considers the performance in the three split disciplines and the two transitions and the complex relationship between the components (1). Pacing describes how an athlete distributes work and energy and plans how to execute his race (3). The pacing considers also the changes in speed during a single discipline (3).

The interest in pacing strategies dates back as far as a century, with researchers interested in understanding how different energy systems contribute to the performance of athletes and how the tests should be carried out to obtain a better performance (4). Over the past few years, this mechanism has been widely investigated in athletes of different running events, such as the 5 km run (5), the 10 km run (6), half marathon, marathon and ultramarathon running events (7, 8), and in different swimming events (9–11) as it is identified as a determining factor in the performance of athletes. In ultra-marathoners, pacing has been studied as an essential part of the athletes’ performance (12, 13). In swimming, the analysis of pacing has been performed for short (14), and long distances to better understand the swimmer's effort and performance during the race (15). As for cycling, longer distances require a better management of power, energy costs and velocity (16). Therefore, pacing constitutes an essential part of a cyclist performance (17).

**Figure 1:**
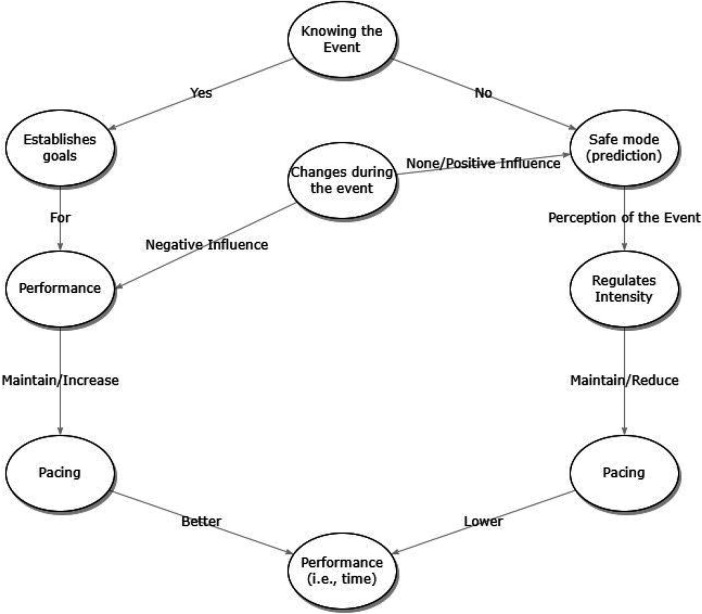
Theoretical representation of the “tele anticipation” model, proposed by Ulmer in 1996 (18, 19).

Different pacing strategies are known, such as negative, all-out, positive, even, parabolic-shaped, and variable pacing (3). Different models were proposed to understand the mechanisms that explain pacing strategies. In 1996, Ulmer proposed one of the first models, called the “tele anticipation” (18, 19). The model is based on communication between the central and peripheral nervous systems. Thus, throughout a race, the athlete's perception of effort and physiological and metabolic changes would keep the activity at a “safe” threshold, contributing to the task being completed without physiological failure (20). That is, it is based on these “perceptions” that the athlete makes adjustments throughout the race, especially in pacing (21). In addition to these factors, athlete characteristics (background, motivation, mood and/or knowledge of the route) have an influence on the adjustments to the exercise intensity (18). Therefore, through this model (proposed in Figure 1), the strategy adopted by the athlete is determined in advance, which does not mean that it cannot be restructured throughout the race since a set of external factors (temperature, altitude, wind speed and presence of opponents) can influence the strategies adopted (3). In addition, as pacing is considered a complex behavior (22), the role of the physiological (heart rate), recovery (quality and quantity) and morphological indicators (body mass) needs to be considered. Especially in multi-day events (23), where the variability of these indicators could influence cognitive aspects and performance output (24).

To date, endurance and ultra-endurance athletes are pushing their limits to the extreme. While one marathon in one day is enough for most athletes, others run seven marathons in seven days (25) or even ten marathons in 10 days (26). Also, ultra-triathletes are pushing their limits. In the past, “IRONMAN® Hawaii” was the ultimate triathlon race (27). Today, triathlon race distances have extended to x-times the IRONMAN® distance (3.8 km swimming, 180 km cycling, 42.195 km running) (28), ending in 30 Ironman-distance triathlons in 30 days (29). While pacing in a single IRONMAN® triathlon is based on splits during a single discipline such as cycling, running (30), or swimming (9–11), the analysis of pacing strategy in a multi-stage triathlon is based on a day-by-day basis (23) and split times of the single disciplines (31).

While the 30 Ironman-distance triathlons in 30 days were the longest official multi-stage ultra-triathlon race to date (29), athletes strive to set new records and new limits. Shortly after the 30 Ironman-distance triathlons, a triathlete set a new record, finishing 33 Ironman-distance triathlons in 33 days (32). Recently, a triathlete completed 40 Ironman-distance triathlons in 40 days (33). Because these athletes recorded all their split times, including some physiological variables such as heart rate, body mass, etc., case studies about their record events were possible. Furthermore, James Lawrence from the USA has already completed 50 Ironman-distance triathlons in 50 days and even 100 Ironman-distance triathlons in 100 days (https://www.ironcowboy.com/conquer-100/) which shows the athletes’ ambition to excel.

In the present case study, we investigated the self-selected pacing of a professional IRONMAN® triathlete who finished 20 Ironman-distance triathlons in 20 days, then 40 Ironman-distance triathlons in 40 days, and finally 60 Ironman-distance triathlons in 60 days. Based on the “tele anticipation” model, the presented model in Figure 1 suggests that the pacing strategy will vary depending on the events for expected, unexpected, predictable, and unpredicted variables and conditions. We analyzed (i) the pacing of the split disciplines of swimming, cycling, and running, and (ii) we compared the performances by Decas (i.e., 10 Ironman-distance triathlons in 10 days).

## Case description

### Ethical approval

This study was approved by the Institutional Review Board of Kanton St. Gallen, Switzerland, with a waiver of the requirement for informed consent of the participant as the study involved the analysis of publicly available data. The study was conducted in accordance with recognized ethical standards according to the Declaration of Helsinki adopted in 1964 and revised in 2013.

### Athlete

The subject is a professional IRONMAN® triathlete, and all his details (training, heart rate, split times, etc.) are recorded on his website (www.raitratasepp.ee/). Details about his anthropometry and training have been described in an earlier case study (33). In all three events, he used the same kind of equipment including a wetsuit for swimming and his preferred bike. He is an elite athlete in self-paced events of multiple Ironman-distance triathlons. In 2018, he completed in Fuerteventura 20 Ironman-distance triathlons in 20 days within 238:52:34 h:min:s. In 2019, he finished 40 Ironman-distance triathlons in 40 days in 444:21:35 h:min:s, and in 2021, he finally completed 60 Ironman-distance triathlons in 60 days in 657:40:09 h:min:s (www.raitratasepp.ee/results/ultra-triathlons). All recorded times are unofficial world records. In 2022, he set an official world record approved by IUTA (International Ultra-Triathlon Association) in five Ironman-distance triathlons in five days in 49:32:49 h:min:s (www.iutasport.com/records/world-records) to be the first triathlete in the world to finish each Ironman-distance triathlon in this version in less than 10 h.

### Data analysis

For 20 Ironman-distance triathlons in 20 days (Event 1), details were from his website (www.raitratasepp.ee/my-self-organized-challenges/20-times-ultra-triathlon-2018-fuerteventura). All details were obtained from his website for 40 Ironman-distance triathlons in 40 days (Event 2) (www.raitratasepp.ee/my-self-organized-challenges/40-times-ultra-triathlon-2019-fuerteventura). For the 60 Ironman-distance triathlons in 60 days (Event 3), he summarized all his data in a blog (www.raitratasepp.ee/blog-en/statistics-by-disciplines-and-days). Details about the logistics, nutrition, and support have been published in an earlier case report (33). The location Fuerteventura did not change between the three events. We used Disciplines – Swimming, Cycling, Running, and Total event time – in the analyses. Additional events data (sleep duration, body mass, cycling heart rate, running heart rate) were also included where available. In particular, heart rate during swimming was unavailable in all events, while all additional data were unavailable in Event 1.

### Statistical analysis

Before all statistical tests, descriptive statistics were calculated as mean and standard deviation. Data distribution normality was verified by the Kolmogorov-Smirnov (KS) test and a visual inspection of histograms and QQ plots. Further analysis included linear regressions with the corresponding correlation coefficients (*r*). All correlation coefficients were interpreted as small, *r* = 0.10–0.29; moderate, *r* = 0.30–0.49; and large, *r* = 0.50–1.0 (Cohen, 1988). Linear regressions were applied on a mean daily result for each discipline within the event (i.e., swimming, cycling, running and total time). Moreover, the same linear regressions were performed on mean additional events data (i.e., sleep duration, body mass, and heart rate during cycling and running). To test differences between Events (independent) and Decas (paired) within the same discipline, Student's *t*-test (2 groups) and one-way ANOVAs (3 or more groups) were used. The same Student's *t*-test and ANOVAs were used to test differences between Events (independent) and Decas (paired) for additional events data. For all ANOVAs, the *post hoc* Bonferroni test was performed, while the alpha level was set at *p* < 0.05. For all ANOVAs and *T*-test, eta squared (*ŋ*^2^) was calculated as effect size, where the values of 0.01, 0.06, and above 0.14 were considered small, medium, and large, respectively (34). All statistical tests were performed using Microsoft Office Excel 2017 (Microsoft Corporation, Redmond, WA, USA) and SPSS 26 (IBM, Armonk, NY, USA).

## Results

The mean Deca results for all three events shown in total and separately for swimming, cycling, and running were presented in Table 1.

**Table 1:** Mean deca times for all three events and disciplines (h:min:s).

Event	Deca	Swimming	Cycling	Running	Total
1	1	1:11:41	6:26:31	3:46:59	11:49:42
	2	1:12:18	6:44:00	3:44:41	12:03:33
	Average	1:12:00	6:35:16	3:45:50	11:56:37
2	1	1:14:27	6:07:41	3:34:01	11:17:35
	2	1:15:17	6:14:41	3:28:15	11:15:40
	3	1:15:30	6:04:00	3:17:26	10:51:28
	4	1:17:20	6:07:21	3:23:56	11:01:24
	Average	1:15:39	6:08:26	3:25:55	11:06:32
3	1	1:19:31	5:59:59	3:19:39	10:54:42
	2	1:23:10	6:18:20	3:19:15	11:20:06
	3	1:20:52	5:56:17	3:14:06	10:47:54
	4	1:20:38	5:53:32	3:12:40	10:42:34
	5	1:21:05	5:57:40	3:09:33	10:44:29
	6	1:21:46	6:07:24	3:12:40	10:58:10
	Average	1:21:10	6:02:12	3:14:39	10:54:39

Deca = 10 Ironman-distance triathlons in 10 days.

**Figure 2:**
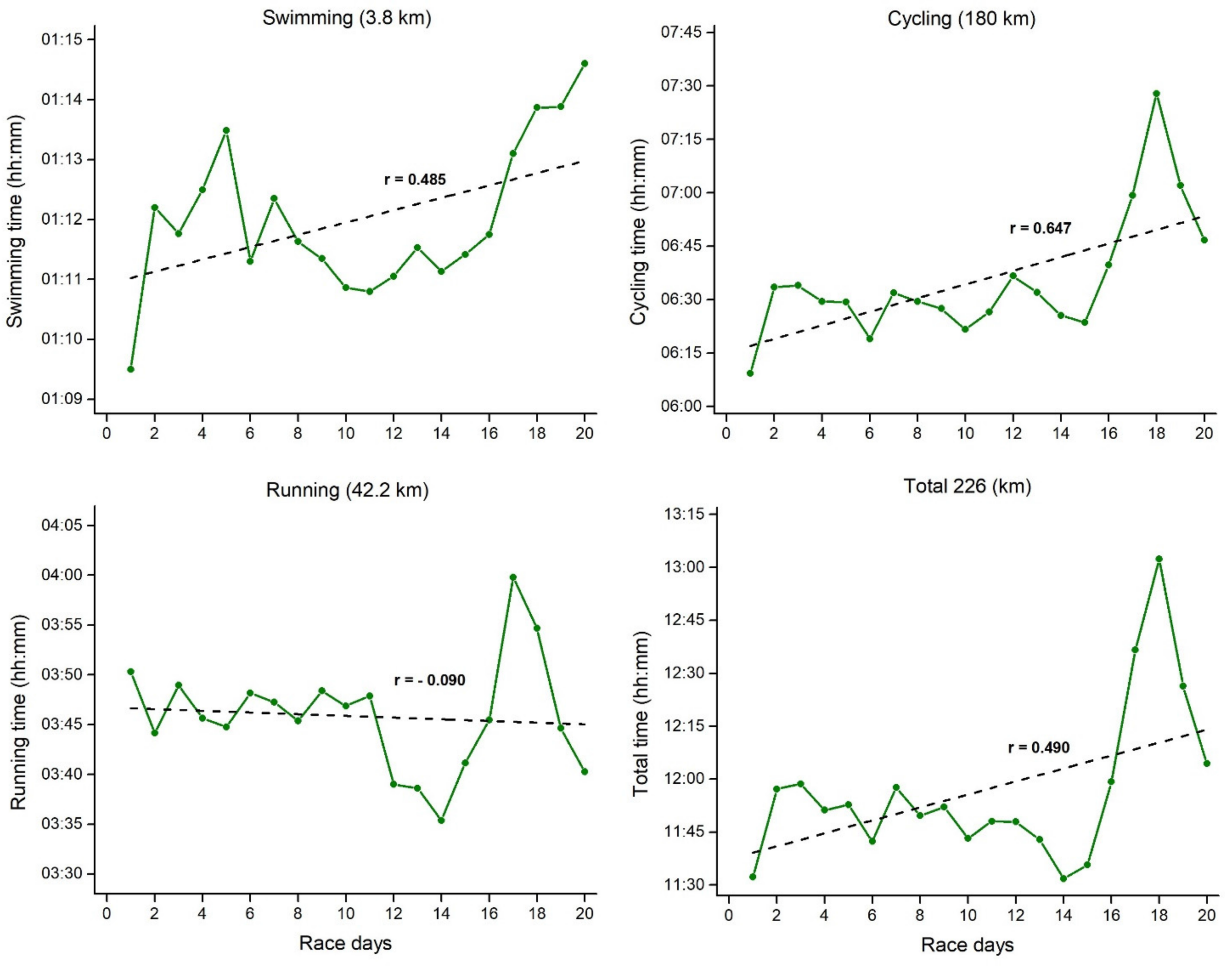
Mean daily results per discipline for event 1 (20 ironman triathlons in 20 days) with the corresponding linear regressions and correlation coefficients.

**Figure 3:**
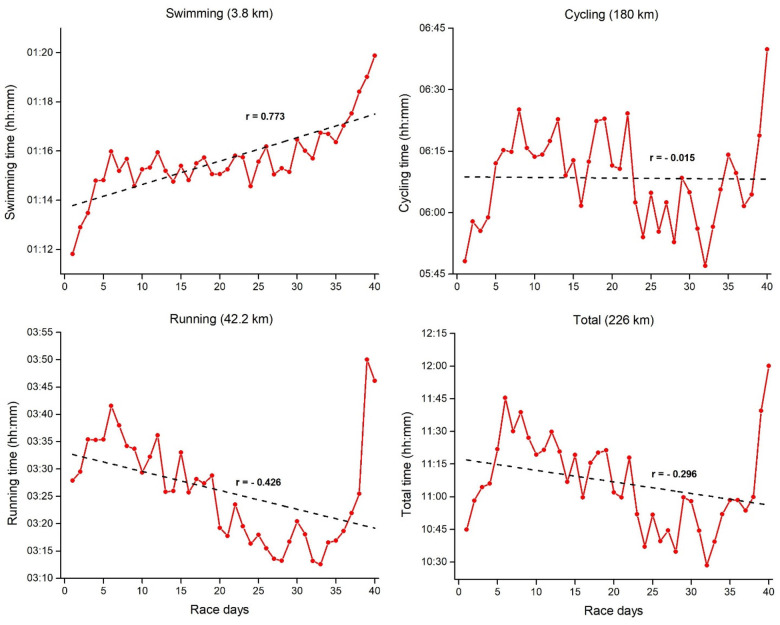
Mean daily results per discipline for event 2 (40 ironman triathlons in 40 days) with the corresponding linear regressions and correlation coefficients.

**Figure 4:**
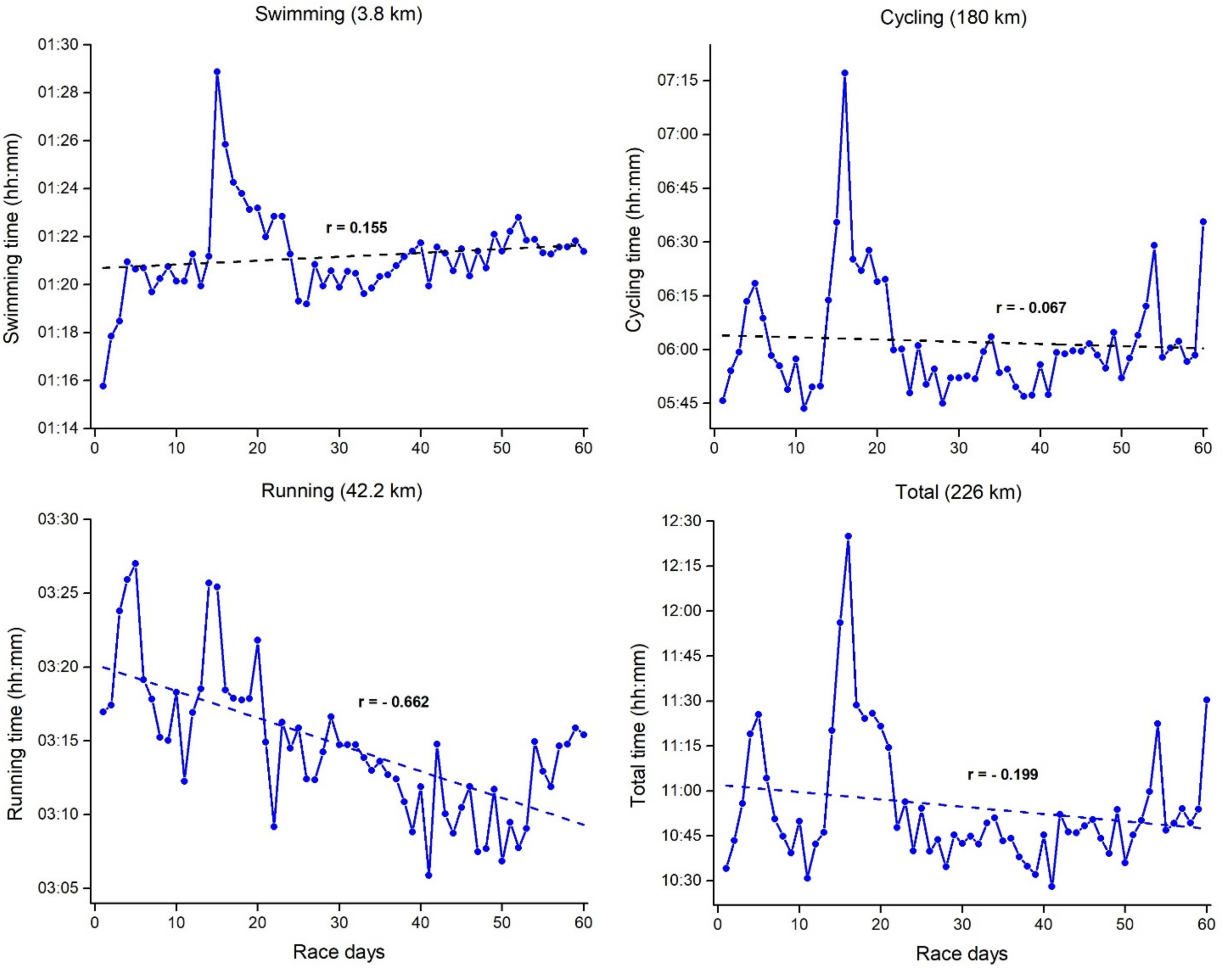
Mean daily results per discipline for event 3 (60 ironman triathlons in 60 days) with the corresponding linear regressions and correlation coefficients.

Figures 2–4 represents mean daily results per discipline for Event 1, 2 and 3 with the corresponding linear regressions and correlation coefficients. Swimming, cycling, and total daily results showed a positive and moderate-to-large correlation with the race days, thus indicating a slowdown as Event 1 progressed. Contrary to that, running results showed no correlations with the race days, indicating consistent running results through all 20 days. For the three disciplines in Event 2 (Figure 3), a higher variability was verified across the race days, with different trends through the linear regression results. Swimming showed a significant positive correlation with the race days, thus indicating a slowing down in this discipline as Event 2 progresses. In contrast, cycling results showed no correlations with the race days, thus indicating consistent results through all 40 days. Contrary to Event 1, running and total time in Event 2 showed a moderate negative correlation with the race days. It means a decrease in the finish time over days. Visually, it is possible to verify an increase in running time on race day 40. Finally in the Event 3 (Figure 4), swimming showed a slight positive correlation with the race days, while cycling results showed no correlations with the race days, indicating relatively consistent results through all 60 days. Similar to Event 2, running and total time in Event 3 showed a large and small negative correlation with the race days, respectively.

**Figure 5:**
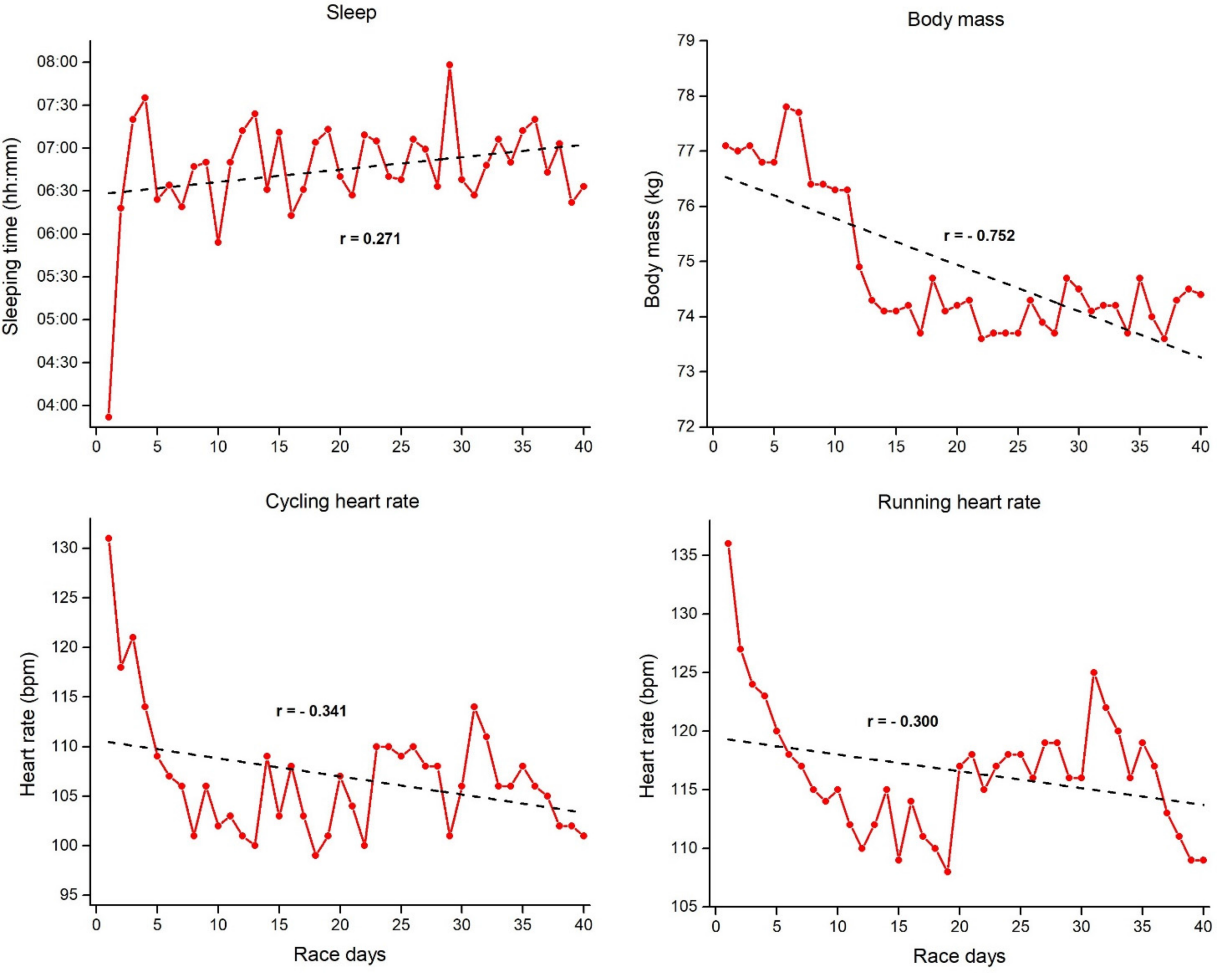
Mean daily results of sleep duration, body mass, cycling, and running heart rate for event 2 (40 ironman triathlons in 40 days) with the corresponding linear regressions and correlation coefficients.

**Figure 6:**
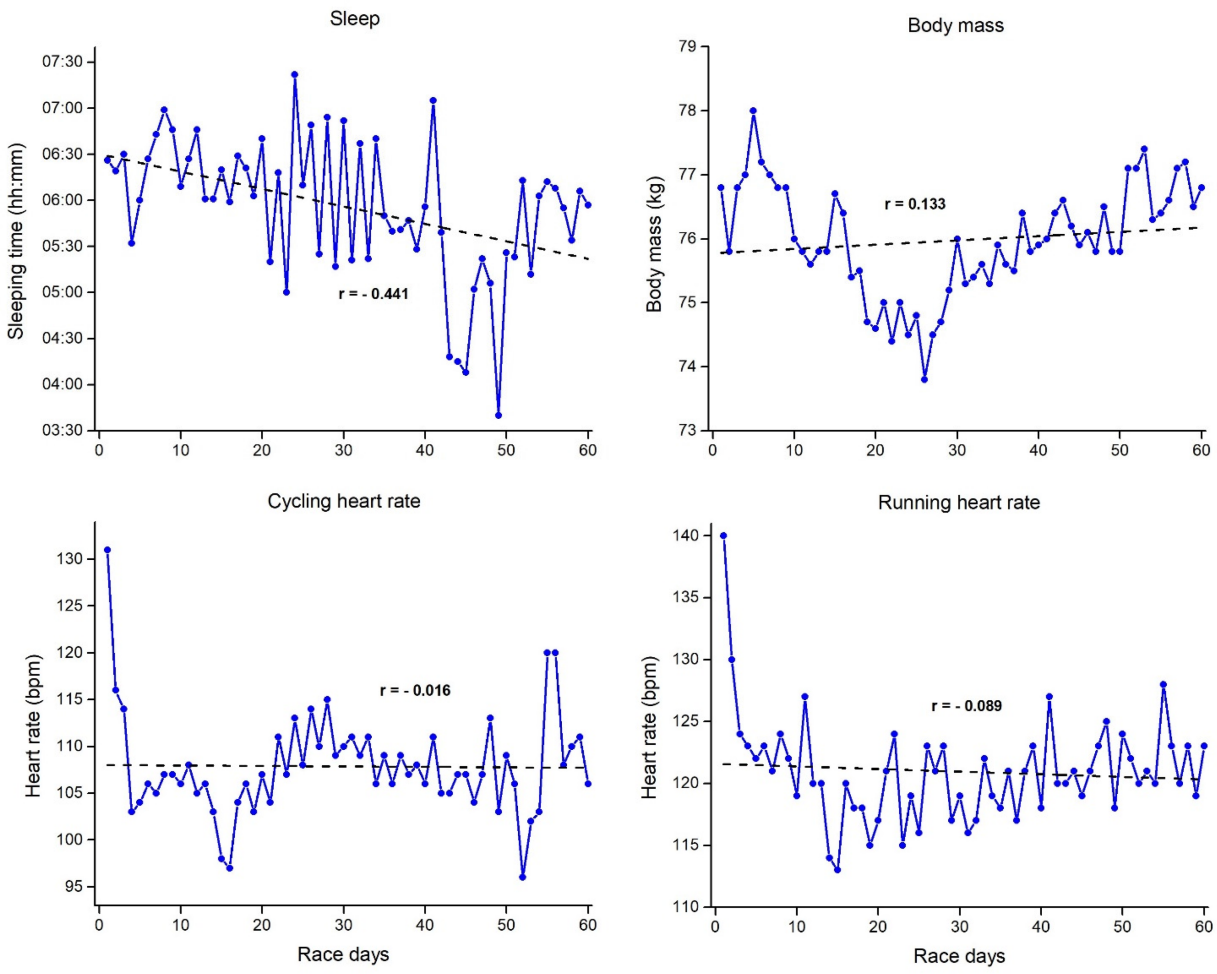
Mean daily results of sleep duration, body mass, cycling, and running heart rate for event 3 (60 ironman triathlons in 60 days) with the corresponding linear regressions and correlation coefficients.

Figures 5, 6 represent the mean daily sleep duration, body mass, cycling, and running heart rate for Event 2 and Event 3 with the corresponding linear regressions and correlation coefficients. Sleep duration showed a slight positive correlation with the race days, thus indicating a slight increase in sleep duration as Event 2 progressed. In contrast, body mass showed negative correlations with the race days, indicating a constant body mass loss through all 40 days. However, despite the negative correlation, the body mass loss was not constant. The data showed a decrease in body mass until day 15, after that, the body mass was constant. Finally, both running and cycling mean heart rates showed a moderate negative correlation with the race days. Contrary to Event 2, in the Event 3 sleep duration showed a moderate negative correlation with the race days, thus indicating a slight decrease in sleep duration as Event 3 progresses. Body mass showed small positive correlations with the race days, indicating a slight trend to weight gain, while both running and cycling mean heart rates showed no correlation with the race days.

Table 2 presents significant main effects for Events (independent) and Decas within the same discipline (paired), for all triathlon disciplines and additional data.

**Table 2:** Significant main effects for events (independent) and decas within the same discipline (paired), for all triathlon disciplines and additional data.

	Main effects	Event 1 (Deca 1–2)	Event 2 (Deca 1–4)	Event 3 (Deca 1–6)	Deca 1 (Events 1–3)	Deca 2 (Events 1–3)	Deca 3 (Events 2–3)	Deca 4 (Events 2–3)
Swimming	T/F	1.041	17.251	6.779	81.809	111.620	12.178	6.792
	*η* ^2^	0.057	0.657	0.386	0.858	0.892	0.796	0.548
	*p*	0.325	**<0** **.** **001**	**<0**.**001**	**<0.001**	**<0.001**	**<0**.**001**	**<0**.**001**
Cycling	T/F	2.814	1.711	4.185	17.212	6.445	−1.857	−2.874
	*η* ^2^	0.305	0.016	0.279	0.560	0.323	0.083	0.179
	*p*	**0.020**	0.210	**<0**.**001**	**<0.001**	**<0.001**	0.080	**0**.**010**
Running	T/F	−1.075	8.256	16.417	129.349	55.169	−2.721	−2.821
	*η* ^2^	0.060	0.478	0.603	0.905	0.803	0.163	0.173
	*p*	0.310	**<0**.**001**	**<0**.**001**	**<0.001**	**<0.001**	**0**.**014**	**0**.**019**
Total	T/F	1.805	3.095	5.817	29.209	9.526	−1.120	−2.493
	*η* ^2^	0.153	0.256	0.350	0.684	0.414	0.032	0.141
	*p*	0.105	**0**.**044**	**<0**.**001**	**<0.001**	**<0.001**	0.277	**0**.**032**
Sleeping time	T/F	/	1.972	7.061	/	/	2.589	5.572
	*η* ^2^	/	0.180	0.440	/	/	0.150	0.450
	*p*	/	0.142	**<0**.**001**	/	/	**0**.**022**	**<0**.**001**
Body weight	T/F	/	70.663	24.250	/	/	−3.200	−9.862
	*η* ^2^	/	0.910	0.729	/	/	0.212	0.719
	*P*	/	**<0**.**001**	**<0**.**001**	/	/	**<0**.**001**	**<0**.**001**
Cycling heart rate	T/F	/	3.901	1.813	/	/	−2.202	−1.467
	*η* ^2^	/	0.302	0.168	/	/	0.113	0.054
	*P*	/	**<0**.**001**	0.193	/	/	**0**.**041**	0.160
Running heart rate	T/F	/	8.582	4.781	/	/	−2.405	−1.628
	*η* ^2^	/	0.488	0.347	/	/	0.132	0.065
	*p*	/	**<0**.**001**	**0**.**014**	/	/	**0**.**033**	0.129

T/F - T or F ratios for the *T*-test and ANOVA; *η*^2 −^ eta squared; The bold values indicate the statistical significant *p* value (<0.05).

**Figure 7:**
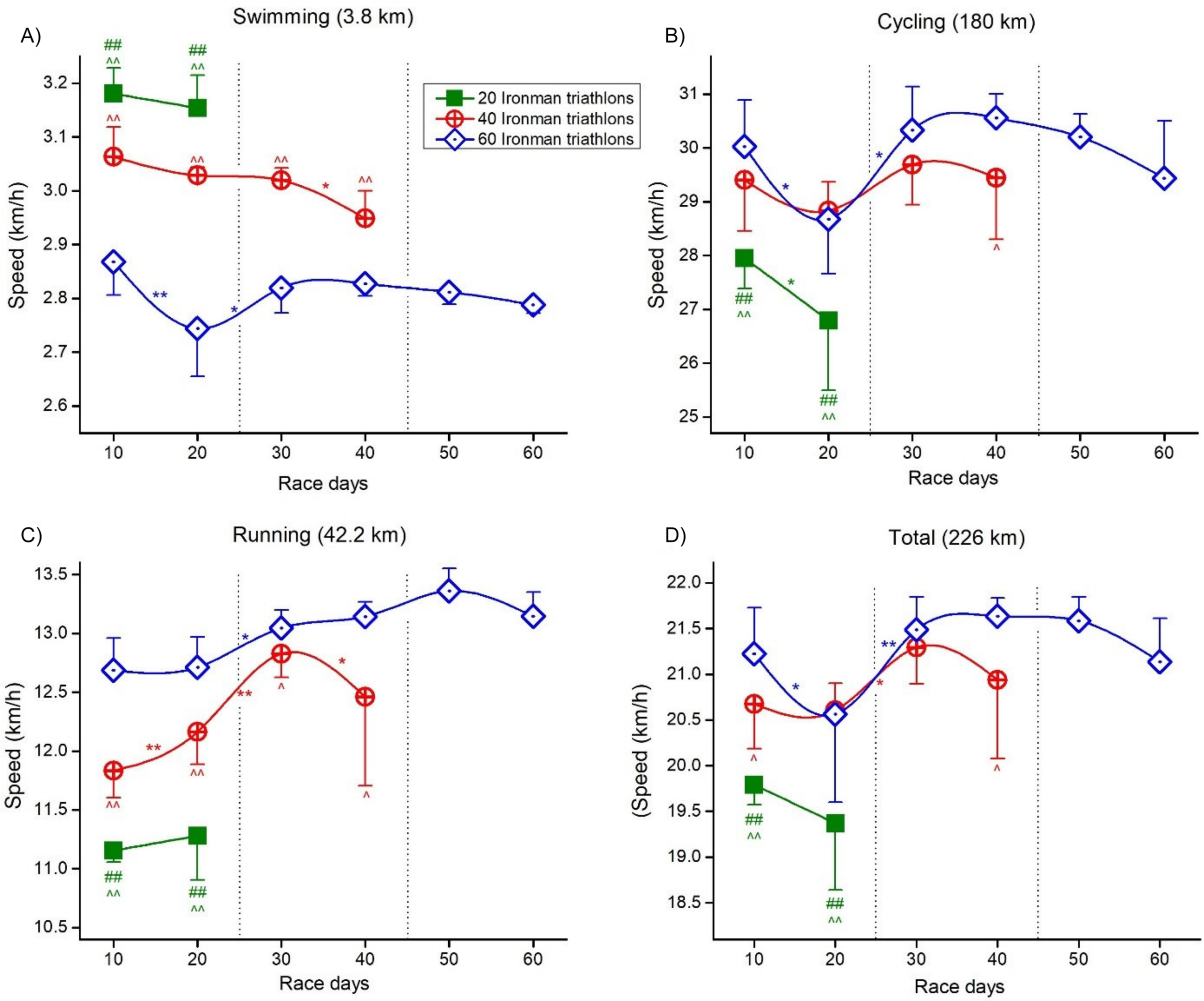
Differences between events and deca intervals for each triathlon discipline. Speed and race days plots: **(A)** Swimming; **(B)** Cycling; **(C)** Running; **(D)** Total. * and ** significant difference between Deca intervals at *p* < 0.05 and *p* < 0.01, respectfully. ^##^significant difference from Event 2 at *p* < 0.01; ^ and ^^ significant differences from Event 3 at *p* < 0.05 and *p* < 0.01, respectively.

**Figure 8:**
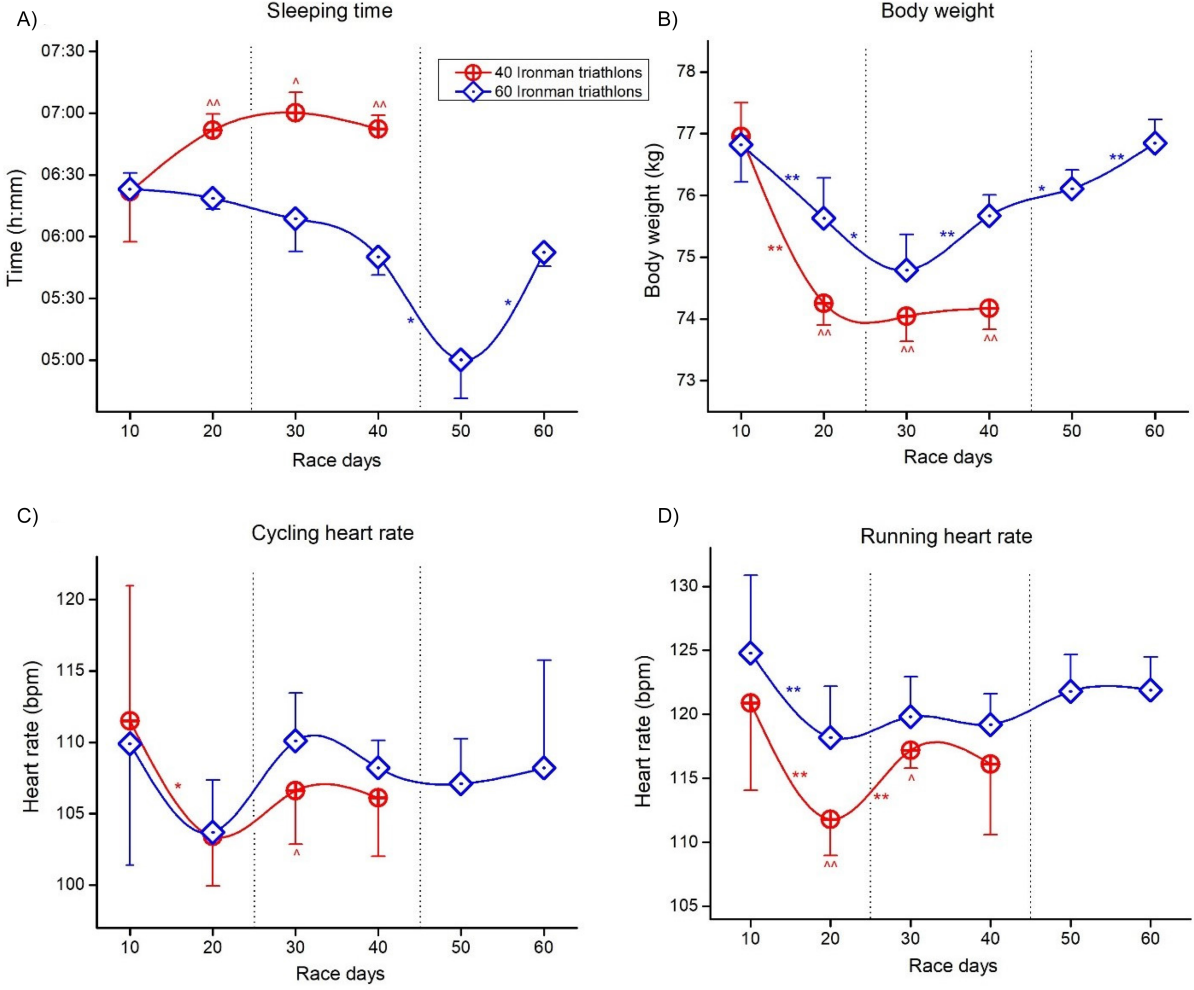
Differences between events and deca intervals for each triathlon discipline. **(A)** Sleep time; **(B)** Body weight; **(C)** Cycling heart rate; **(D)** Running heart rate. * and ** significant difference between Deca intervals at *p* < 0.05 and *p* < 0.01, respectfully; ^ and ^^ significant differences from Event 3 at *p* < 0.05 and *p* < 0.01, respectively.

Figure 7 (for all triathlon disciplines) and Figure 8 (for additional data) present mean data and the post-hoc results of the aforementioned analyses.

## Discussion

In the present case study, we analyzed the pacing of the split disciplines of swimming, cycling, and running. We compared the performances by Decas (i.e., 10 Ironman-distance triathlons in 10 days) in the self-selected pacing of a professional triathlete who finished 20 Ironman-distance triathlons in 20 days, then 40 Ironman-distance triathlons in 40 days, and finally 60 Ironman-distance triathlons in 60 days. We expected that the pacing strategy would vary depending on the events for expected, unexpected, predictable, and unpredicted variables and conditions.

The most important findings in this case study, investigating the self-selected pacing in 20 (Event 1), 40 (Event 2), and 60 (Event 3) Ironman-distance triathlons with physiological variables for Event 2 and Event 3 were: (*i*) swimming splits were fastest in Event 1, (*ii*) cycling and running splits were fastest in both Event 2 and 3, (*iii*) overall times were fastest in Event 3, (*iv*) sleep duration increased during Event 2 but decreased in Event 3, (*v*) body mass decreased in Event 2, but increased in Event 3 and (*vi*) cycling heart rate was similar in both Event 2 and 3 while running heart rate was greater in Event 3.

### Differences in the split and overall times

When comparing split and overall times between Decas for the three events, swimming was fastest in Event 1 and slowest in Event 3, cycling and running were fastest in Event 3 and slowest in Event 1, and overall speed was slowest in Event 2 and fastest in Event 3. The length of the event can explain these disparate findings for the different events. An investigation of 48 male ultra-triathletes competing in five Ironman-distance triathlons in five days (*n* = 14), 10 Ironman-distance triathlons in 10 days (*n* = 25), and 20 Ironman-distance triathlons in 20 days (*n* = 9) showed that the athletes achieved a stable cycling performance independent of the length of the race (31). Still, the cycling split influenced the subsequent running split depending on the length of the race (31). However, another likely explanation could be that he was better trained in 2021 than in 2019 since he was able to set a new world record in five Ironman-distance triathlons in five days in 2022 (www.iutasport.com/records/world-records). Furthermore, he might have had more experience competing over several days. Since he selected the same location in Fuerteventura for all three events, he probably had the best conditions (event site, weather, etc.) for such a challenge.

### Changes in pacing in split and overall times over days

During Event 1, swimming, cycling, and overall times increased, whereas running times remained stable. During both Event 2 and 3, swimming times increased, cycling times remained stable, and both running and overall times decreased. An increase in overall times (i.e., positive pacing) is generally seen in non-stop ultra-cycling performances (8, 35), in single IRONMAN® triathlons (30) and also in multi-stage Ironman-distance triathlons (23, 29). In some instances, athletes can achieve an even pacing in non-stop ultra-endurance performances (7) and multi-stage races (29, 32). An athlete who finished 33 Ironman-distance triathlons within 33 consecutive days with minor variations in both split and overall race times achieved even pacing. It was assumed that even pacing was most probably due to the high experience of the athlete and the stable environmental conditions (32). Also, a world-class female ultra-triathlete (36) and a paraplegic triathlete (36) competing in both a 5× and 10× Ironman-distance triathlon race were both able to adopt an even pacing. Our athlete showed stable running times during Event 1 and decreasing running and overall race times in Event 2 and 3. This means that he is able to sustain a negative pacing in a multi-day Ironman-distance triathlon, which is most probably due to his great experience in ultra-running (www.raitratasepp.ee/). In 2020, he ran 20 marathons in 20 consecutive days where all marathons were below 3 h (www.raitratasepp.ee/my-self-organized-challenges/20-marathons-in-20-consecutive-days-2020-estonia).

Most probably, a high experience in ultra-endurance performance is needed for the best pacing strategy in an ultra-endurance race (37, 38). The fastest ultra-runners in “Spartathlon” (i.e., 246 km ultramarathon race) showed fewer changes in running speed than slower runners (38). For IRONMAN® triathletes, a fast personal best time in a short race such as an Olympic distance triathlon is important for a more extended race (39). For ultra-triathletes, the previous experience seemed necessary for performance in longer ultra-triathlon races where the personal best times of shorter races were important, but not the number of previously finished races (37). Similarly, speed during training for all split disciplines is related to overall IRONMAN® race times (40). However, numerous variables such as training quantity, type of periodized plan, muscle fiber distribution and other physiological aspects influence triathlon race performance.

The athlete is both a runner and a triathlete where running is his best discipline. During his 20 marathons in 20 days, the fastest marathon was on the last day with a time of 2:47:07 h:min:s (www.raitratasepp.ee/my-self-organized-challenges/20-marathons-in-20-consecutive-days-2020-estonia). The athlete is not only a fast IRONMAN® triathlete, but he is also a relatively fast marathon runner. He has run in 2020 20 marathons in 20 days where each marathon was below 3 h (www.raitratasepp.ee/blog-en/20-marathons-in-20-days-under-3-hours-numbers-and-statistics). In 2021, he ran 20 marathons in 10 days, where again, each marathon was below 3 h (www.raitratasepp.ee/blog-en/20-marathons-in-10-days-all-under-3-hours-numbers-and-statistics).

His high experience in both multi-stage running and multi-stage Ironman-distance triathlon probably explains why he improved his running times during the events (23). A study investigating ultra-marathoners showed that high-performance ultra-runners made less substantial speed reductions during the race than low-performance runners (41). A conservative pacing strategy with a lower running speed at the beginning and a higher running speed towards the end of the event seems to be the best strategy (41). Similarly, for ultra-marathoners competing in the Ultra-trail du Mont Blanc (UTMB®), even pacing throughout the race correlated with faster finishing times (7).

In an IRONMAN® triathlon, both cycling and running are decisive for overall race performance (42). Although the cycling split is the discipline with the most significant influence on overall IRONMAN® race times (43, 44), running seems to be the most important split discipline in both single IRONMAN® triathlons (45) and ultra-triathlons longer than the single IRONMAN® distance (46). It has been shown that ultra-triathletes should distribute their energy among swimming, cycling, and running depending on the race distance (28). In IRONMAN® triathletes, the fastest ones were the fastest in the running; therefore, race tactics in an IRONMAN® triathlon should focus on saving energy during swimming and cycling for a fast-running split (45). A study investigating the importance of the split disciplines in ultra-triathlons of different distances from Double Iron (7.6 km swimming, 360 km cycling, 84.4 km running) to Deca Iron (38 km swimming, 1,800 km cycling, 422 km running) ultra-triathlon showed that the fastest ultra-triathletes spent more time in swimming and cycling and less time in running, highlighting the importance of the role of running for the overall race performance (46). The present results also corroborate these literature findings, as the swimming split times in Event 2 and 3 were higher than in Event 1. However, the total race times were lower. We also need to consider that energy conservation is usually a balance whereby athletes consume gels, water, and carbohydrate blends during the cycling split. Here, it is not necessarily conserving energy but topping up existing or depleted nutritional storage.

We found that swimming splits were fastest in Event 1 whereas swimming had no influence on the longer event distances. Swimming is rarely a good predictor of overall race success in triathlon disciplines. It generally relates as a poor correlate. In IRONMAN 70.3®, running and cycling splits were more predictive than swimming for overall race times (47). When race distances from Sprint to IRONMAN® triathlon were considered, swimming was only predictive in Sprint- and Olympic-distance triathlon, whereas cycling was the most important split discipline in IRONMAN 70.3®, and running in the full IRONMAN® distance (47), confirming our consideration that the present athlete as a fast marathoner was able to achieve these outstanding performances.

### Differences in sleep duration

Daily sleep duration increased over all days during Event 2. During Event 3, daily sleep duration decreased, which was in agreement with a case study of a cyclist in a simulated 10,000 km tour (48). Nedelec et al. (48) observed a decrease from 7 h:min to 5:13 h:min sleep duration throughout this tour, where the cyclist completed 10,358 km in 24 days (daily distance ∼432 km, cycling time 16 h:min, average speed ∼27 km/h). Although we noticed a decrease in sleep duration in Event 3, numerous studies implicate that sleep deprivation should not affect aerobic performance (49–51). Also, aerobic exercise can eliminate sleep deprivation's negative effects (52). However, in a 3-day ultra-endurance triathlon, the total sleep duration was reduced and showed a significant and negative correlation to exercise performance, where sleep loss was associated with slower performance (24).

Nonetheless, sleep management seems to have an influence on ultra-endurance performance. In ultra-marathoners competing in the “North-Face Ultra-Trail du Mont-Blanc,” most of the finishers were aware of the importance of sleep management strategies and increasing sleep duration some nights before the race, which appeared to be the most relevant strategy to improve race performance (53). Sleep deprivation during the “North-Face Ultra-Trail du Mont-Blanc” has a negative effect on cognitive performance in ultra-marathoners (54). Overall, athletes competing in ultra-endurance competitions need to develop a good sleeping strategy (53, 55). Since sleep increased during Event 2 but decreased during Event 3, we assume that 60 Ironman-distance triathlons in 60 days are the limit and more days would lead to an impaired performance or even a failure of a record attempt. However, considering the “North-Face Ultra-Trail du Mont-Blanc”, trail running requires a different biomechanical approach to running and, therefore, could be argued that it is more taxing and fatiguing compared to a typical road running style triathlon and ultra-triathlon.

### Differences in changes in body mass over time

In both Event 2 and 3, the athlete started with the same body mass of ∼77 kg. In both events, body mass initially decreased until day 20 in Event 2 and day 30 in Event 3. Body mass remained low in Event 2 but increased in Event 3 after day 30 to reach the initial value of ∼77 kg by the end. A decrease in body mass generally occurs during a single IRONMAN® triathlon (56–58) and also during multi-stage Ironman-distance triathlon events (23, 32, 59). The decrease in body mass during a single IRONMAN® triathlon is most likely due to dehydration (60) and body water loss (56, 57) but can also be due to a loss in solid mass (58, 61). This loss in solid mass occurs especially of the lower limbs (57, 61) due to glycogen depletion in the leg muscles (57, 58). The decrease or increase in body mass during a multi-stage event such as a multi-stage Ironman-distance triathlon is most likely due to a change in both solid mass and total body water. While the decrease in body mass during such an ultra-race is most likely due to a reduction in fat mass (23, 59, 62) and muscle mass (63), the increase in body mass in the second half of the Event 3 is most likely due to fluid overload and body water retention (23, 62, 64). Fluid intake seems to be related to fluid overload. It has been shown that overdrinking leads to limb swelling (65).

Changes in body mass would also be influenced by what was consumed, if anything, during the bicycle segment, given that the bicycle segment is generally considered the most practical time to consume. Therefore, body mass would be influenced by what was consumed and the relative quotient of intensity to calories consumed. This also opens further analysis as the type of substrate consumed would influence body mass (i.e., fructose, sucrose, etc.). Essentially, it is highly variable.

However, changes during such races are not always consistent. A study investigating eight male triathletes competing in the first Deca Iron ultra-triathlon in history in the version of one Ironman-distance triathlon per day showed that these athletes lost 3 kg of body fat. In contrast, skeletal muscle mass, mineral mass, and body water remained unchanged during these 10 days (59). Interestingly, the most extensive changes occurred during the first day of the event, with a significant decrease in body mass and body fat in addition to a significant increase in total body water (59). Indeed, although the body mass had decreased significantly during Event 2, there was no negative impact on sports performance since the race time improved. Little is known regarding changes in body mass during long-distance swimming. One swimmer covering 26 km in an open-water swim lost 1.1 kg body mass during 9 h of non-stop swimming (66). In a sample of 20 male swimmers competing in “Lake Zurich Marathon Swim” covering 26 km, body mass decreased significantly by 0.5 kg, mainly due to a decrease in skeletal muscle mass (67).

The type of exercise also seems to be of importance. While ultra-running leads to a decrease in both skeletal muscle mass and fat mass (68), ultra-cycling leads to a decrease in fat mass but not in skeletal muscle mass (69). The observed significant variation of body mass in the present case study indicates a difficulty in achieving an equal energy balance, i.e., matching the number of calories consumed from drinks and food with the amount burnt during energy expenditure (physical activity, basal metabolic rate, and thermogenic effect of food).

For swimming, different predictors have an influence on swimming performance such as body composition (70), training (71), and equipment such as using a wetsuit (72, 73). It is well-known that wearing a wetsuit improves swimming performance (74, 75) especially in triathletes (76). Male swimmers seem to profit more in shorter long-distance swimming events than in longer ones (75). Therefore, our athlete as a fast runner but rather weak swimmer with swim times of longer than 1:10 h:min seems to profit from wearing a wetsuit.

### Differences in changes in mean heart rate during cycling and running

Heart rate during cycling and running decreased over days during Event 2 but remained stable in Event 3 during the whole event. Furthermore, heart rate was overall higher in Event 3, which was higher in running than in cycling. A potential explanation could be that he was “pushing harder” in Event 3, where he was well prepared after having successfully completed Event 1 and Event 2. Also, sleep deprivation might increase his heart rate (77). Furthermore, environmental influences such as heat could have had an effect. An influence on the heart has been well described in ultra-endurance performances such as ultra-marathon running (78, 79). Performance and heart rate are directly linked (80). In ultra-endurance cyclists, both power and heart rate decrease during a race (81, 82). A multi-stage ultra-marathon over 64 consecutive days leads to a significant increase in left ventricular mass with no signs of adverse cardiovascular modeling (79). A single ultra-marathon run leads to changes in resting heart rate, blood pressure, and pulse pressure, all associated with cognitive function impairments (78). Mental fatigue leads to an impairment of cognitive performance where also endurance performance is impaired by mental fatigue (83).

Longer ultra-endurance performances seemed to have a negative effect on the heart (84). In cyclists completing a 21-day cycling ride, arrhythmia rates (supraventricular and ventricular arrhythmias) increased with no changes in the cardiac structure (85). The observed decrease in heart rate in the participant of the present case study might be due to the effect of endurance exercise on the autonomous nervous system, increasing parasympathetic and decreasing sympathetic activity (86). In this context, endurance exercise increases the quantity of blood that the heart pumps in each beat, and consequently, it decreases the submaximal heart rate (87). This might be due to improved left ventricular contractibility and increased stroke volume.

### Limitations

One of the potential limitations of this study could be the use of the group design statistical approach for a single subject. However, it was statistically appropriate to perform parametrical statistical analyses due to the large number of data (i.e., separate days of data). Also, it was not possible to perform more complex analyses, such as two-way ANOVA, due to an unequal number of days in three events. Considering that the events were held at a 4-year interval (2018–2021), it is possible that a change in physical fitness level also impacted the results, besides the events’ distances. Physiological data for all events was unavailable from the athlete (88). Differences in physiological capabilities and a more mature cardiovascular system would also, one would assume, influence the output. The athlete's motivation was not considered, which might have influenced the results (89). Intake of solid food and fluids was not available (90) to estimate a potential fluid overload with influence on body mass. Energy intake and energy expenditure were not determined to estimate an energy deficit (56, 89, 90). We did not consider environmental conditions which might influence the athlete's performance (33, 35). The lack of heart rate data in the swimming section is a further limitation.

### Practical applications, and implications for future research

Overall, this case study shows that performing a daily Ironman-distance triathlon is possible over consecutive days. This case study might provide an example of the effect of daily strenuous exercise lasting many hours (∼10.5–12 h) on heart rate, sleep duration and body mass. Among these three parameters, body mass and heart rate decreased during the first 20 days in both 40 and 60 Ironman-distance triathlons. In contrast, sleep duration did not show a common pattern between these two triathlon formats. The decrease of body mass and heart rate as chronic adaptations to exercise have been well known; however, the findings highlighted the need of sleep management in ultra-endurance athletes. Therefore, the findings of this case study have implications for both research and practice; they enhance our understanding of daily strenuous exercise lasting many hours and provide practical information for IRONMAN® triathletes competing in multiday events. In future case studies, the including of heart race during swimming would be of great interest. Future case studies might investigate the pacing of James Lawrence during his 100 Ironman-distance triathlons in 100 days held in 2021.

## Conclusion

In a professional IRONMAN® triathlete finishing 20, 40, and 60 Ironman-distance triathlons in 20, 40, and 60 days, respectively, split performances and physiological changes such as body mass and heart race differed depending upon the duration of the events. These results highlight the complexity of the study of athlete performance and reinforce the importance of using pacing strategies to better understand individual behavior. It seems that an athlete starting such a challenge might become faster with increasing duration of the challenge. Future athletes intending to complete more Ironman-distance triathlons in a row may profit from the insight in this pacing strategy analysis. Energy intake and energy output and changes in body composition need more investigation in these multi-day challenges in order to properly master energy balance and body composition.

## Data Availability

The raw data supporting the conclusions of this article will be made available by the authors, without undue reservation.
